# The role of government’s ‘Owned Media’ in fostering cultural inclusion: a case study of the NSW Department of Education’s online and social media during COVID-19

**DOI:** 10.1177/1329878X20968291

**Published:** 2021-02

**Authors:** Lauren Gorfinkel, Tanya Muscat, Sue Ollerhead, Alice Chik

**Affiliations:** Macquarie University, Australia

**Keywords:** cultural diversity, education, government–stakeholder communication, home–school communication, inclusion, listening, organisational communication, representation, social media, voice

## Abstract

This article examines government approaches to public communications at the time of the coronavirus disease 2019 (COVID-19) pandemic, with a focus on how one state government body, namely, the New South Wales (NSW) Department of Education in Australia, has engaged with key stakeholders at a time when home–school communications has been heavily impacted by COVID-19. Through analysis of the Department’s ‘owned’ online communications platforms, such as websites, podcasts and social media, the article specifically focuses on how the Department has represented and invited engagement among its culturally and linguistically diverse (CALD) stakeholders with a view to understanding the extent to which it has been able to create a sense of connection and belonging for parents and caregivers. It shares examples of positive practice by the Department as well as suggestions for further research that may help uncover best practices for multicultural and multilingual government–stakeholder engagement.

## Introduction

This article examines government approaches to public communications at the time of the coronavirus disease 2019 (COVID-19) pandemic, with a focus on how one state government body, namely, the New South Wales (NSW) Department of Education in Australia, has publicly engaged with key stakeholders at a time when home–school communications has been heavily impacted by COVID-19. Through analysis of the Department’s ‘owned’ online communications platforms, such as websites, podcasts and social media, the article specifically focuses on how the Department has represented and invited engagement among its culturally and linguistically diverse (CALD) stakeholders with a view to understanding the extent to which it has been able to create a sense of connection and belonging for parents and caregivers. As the public body that oversees the largest education system in Australia (also one of the largest education systems in the world), and the largest single organisation in Australia across public and private sectors (Bagshaw and Nicholls, 2016), the NSW Department of Education offers a pertinent case for examining concepts of organisational communication, listening and voice, as well as representation and inclusion in the context of this global pandemic. It shares pertinent examples of positive practice by the Department while also suggesting that there is room to grow in terms of more consistent engagement across cultures and languages which may foster a greater sense of inclusion. Suggestions for further research that may help uncover best practices for cross-cultural engagement in the context of government and other organisation’s owned media are also provided.

The COVID-19 pandemic, which began in China in late 2019 and spread around the world in 2020, has had a major impact on government bodies, requiring urgent attention to the way they communicate with citizens and stakeholders. While the need for effective government public communication has been most obvious in relation to health communications to ensure the safety of the public in terms of protection from the virus and to stop the spread, the need for effective public communications has had implications across all sectors. The education sector has been massively affected by COVID-19 with the suspension of face-to-face teaching for various periods of time for many students across the globe.

In NSW, the public education system is run under the auspices of the NSW Department of Education which operates 2200 public schools for almost 800,000 students across the state and employs more than 49,000 teachers. After a period of debate over whether schools, workplaces and other public spaces should remain open, on 23 March 2020, the NSW Department of Education issued a media release explaining that the NSW Government under Premier Gladys Berejiklian had taken ‘significant new steps to increase restrictions across the state’, and while schools would remain open, parents in NSW were encouraged to keep their children at home. By this time, nearly 30% of children were already being kept out of school by their parents and caregivers (NSW Department of Education, 2020a). The NSW Department of Education in collaboration with public schools as well as the Australian Broadcasting Association (NSW Department of Education, 2020b) began to more actively share learning from home resources for both parents and teachers via their website and social media to support online and offline learning for all students. This arrangement continued until 11 May, when the Premier and Minister for Education announced a managed return to school (NSW Department of Education, 2020c), with public schools returning to regular full-time face-to-face teaching on 25 May 2020 (NSW Department of Education, 2020d). At the time of writing, COVID-19 continues to affect the running of schools with parents restricted from school grounds and most extra-curricular activities involving parents and carers suspended or transferred to an online format. This has meant an analysis of online communication strategies between schools (and the wider school system) and parents and caregivers is more important than ever.

## Why government engagement with CALD stakeholders is important

Since 1973, Australia has had an explicit policy of multiculturalism. The policy ‘supports the rights of all Australians to celebrate, practice and maintain their cultural heritage, traditions and language within the law and free from discrimination’. It also acknowledges that ‘government services and programs must be responsive to the needs of our culturally diverse communities’ and ‘commits to an access and equity framework to ensure that the onus is on government to provide equitable services to Australians from all backgrounds’. It notes that,

the Australian Government is committed to a just, inclusive and socially cohesive society where everyone can participate in the opportunities that Australia offers and where government services are responsive to the needs of Australians from culturally and linguistically diverse backgrounds. (Department of Immigration and Citizenship, 2011 [2007])

Australia’s latest multicultural statement (Department of Home Affairs, 2017) also acknowledges that while English remains the national language ‘and is a critical tool for migrant integration’, knowledge of the diverse languages that migrants in particular bring to the country are beneficial for sparking ‘innovation, creativity and vitality’ and boosting ‘Australia’s competitive edge in an increasingly globalised economy’. Going beyond the rights of just ‘citizens’, in a statement that may also be seen as inclusive of permanent residents and stakeholders living in Australia with other nationality statuses, NSW has its own multicultural legislation that requires ‘All institutions’ to ‘recognise the linguistic and cultural assets in the population of New South Wales as a valuable resource and promote this resource to maximise the development of the State’ (Multicultural NSW Legislation Amendment Bill, 2014).

In the school context, engagement with CALD communities is of particular significance for the NSW state education system, particularly in a multilingual city like Sydney, the capital of NSW, which has long been Australia’s major immigrant-receiving city. According to the NSW Government’s Centre for Education Statistics and Evaluation (2019), in March 2019, 35.9% or 291,544 primary and secondary students of the 811,802 NSW government school students overall, came from homes where languages other than English were spoken. This was an increase of 9012 students from language backgrounds other than English (LBOTE) from 2018. Speaking 240 different language backgrounds, nearly 60% of all LBOTE students were located in Sydney-West, Sydney-South or Sydney-South West, with only one language background (Macedonian) having a significant concentration of students outside the Sydney metropolitan area. Across all Sydney schools, 55.3% of the students were from LBOTE. Sydney-West had the highest percentage of LBOTE backgrounds in NSW, representing 69.9% of the 90,045 students enrolled at schools. Of these students, the majority had an Indian, Arabic or Chinese language background. As for 8830 ‘new arrival students’ who had been in Australian for less than 6 months, based on data collected through the English as an Additional Language or Dialect (EAL/D) annual census conducted in June 2018, 137 different languages were spoken by students, with Arabic (12.2%), Indian languages (27.6%) and Chinese languages (12.7%) being the most numerous. More than 100 new arrivals spoke Swahili and Kurdish, reflecting a pattern of refugee arrivals from countries with high proportions of speakers in these two languages.

Parents and caregivers, who may include grandparents, siblings, relatives, homestay families and even fellow residents of housing commissions in the case of some students from refugee and asylum seeker backgrounds, play a significant role in supporting the learning of children and helping them to navigate challenges at school. Schools benefit from healthy partnerships with caregivers which result in family and community satisfaction with the school. In particular, caregivers of EAL/D learners, LBOTE students who need targeted English language support to access mainstream Australian schooling, may face unique challenges in relation to the Australian school system. It can be difficult for caregivers to navigate a school system that differs profoundly from that in their home country. School rules, assessments and curricula can seem complicated and obscure. Other challenges may include caregivers feeling that they had no right to participate in school life, or that they did not possess the requisite knowledge of language skills to interact with educators, not to mention that migrants particularly from refugee backgrounds are less likely to have ideal infrastructure and access to technology (Yang et al., 2020). Caregivers of EAL/D students in particular benefit from feeling a sense of belonging and inclusion in Australian society. For these families, schools act as the strongest link to the community, providing an essential source of information, support and social interaction (Premier and Parr, 2019). When this link is broken through school closures or restrictions, home–school relationships can be significantly disrupted and EAL families can be particularly marginalised at such times.

Education systems that recognise the ‘funds of knowledge’ (González et al., 2005) that children bring to their learning environments based on family and community experiences enable all learners to benefit from the rich resources that diverse students bring. This open-to-diversity approach also enables schools to resist the deficit perspectives that dominate discourses about how families of CALD children support their learning at home. Gaining a deeper understanding of the ‘funds of knowledge’ of multilingual children requires dialogic interaction with their families, which may occur online and offline, publicly and privately, and if done well may help to enable a stronger sense of belonging to the school and educational community that makes the sharing of diverse cultural and linguistic knowledge feel safe.

If the onus is on government to provide equitable services to culturally diverse communities, to meet diverse needs and allow for participation from diverse stakeholders, then it is imperative that government educational bodies and schools alike ensure their methods of communication appropriately adapt to, connect and resonate with CALD communities. This makes a fine-grained analysis of the communication strategies of government agencies like the NSW Department of Education an important task. Unlike students and school staff who are present on school grounds, caregivers of students in the NSW public education system, particularly with the impact of COVID-19 restrictions, may rely on online communications to gain connection with the school community and understand the experiences around their children’s education. With official school-based events largely suspended as a result of COVID-19, the online component of home–school communication has become an even more vital link between the school system and homes.

The NSW Department of Education itself has a clear purpose to ‘prepare young people for rewarding lives as engaged citizens in a complex and dynamic society’ and includes among its goals the desire to ‘meet the needs of a growing population’, ensure that ‘community confidence in public education is high’ and that ‘our education system reduces the impact of disadvantage’ (NSW Department of Education, 2019). Its’ values include ‘relationship building’ ‘based on transparency, honesty and mutual respect’ and listening with an open mind (*trust*) as well as respect for ‘diversity and the views and contributions of others’ (*equity*), working ‘openly in partnership with parents, communities and organisations’ (*service*), ‘collaboration and learning with others’, and including ‘the best ideas from everyone in and outside the department’ (*excellence*). The Department’s performance measures include ‘Increased proportion of students reporting a sense of belonging, expectations for success and advocacy at school’ (NSW Department of Education Strategic Plan 2018–2022, 2019). For the government department which guides the tone for and establishes best practice for home–school communication across NSW, enabling positive online engagement which fosters trust and understanding among diverse communities via its own public communication platforms is key to fostering its mandate to facilitate and build an inclusive society.

Despite the significant shift in demographics of school communities over the past decade or more, there remains a large gap in the research on home–school communication of individual schools as well as the communication of the government and non-government educational bodies that oversee the schools in their jurisdiction. Previous studies (Bordalba and Bochaca 2019; Thompson et al., 2015) have found that parents perceived there not to be a necessity to use digital communications where they were able to go to the school grounds and use traditional communication channels. Yet in instances where digital communications become paramount, such as with school closures and restrictions, if parents cannot understand what is happening at their children’s school or do not feel included, they will be unable to support their children’s learning in the best possible way. In this regard, the COVID-19 crisis presents a unique opportunity to investigate how the institution’s use of online and social media functions during a period where parents are unable to visit school grounds and use traditional communication channels. Another concern is the inclusion of culturally diverse voices, which are needed to reflect the realities of the rapidly shifting demographics of schools. The underrepresentation of minority groups in schools’ online and social media communication could have negative ramifications for social inclusion, cohesion, and engagement. This study aims begins to fill these gaps by considering how stakeholders from CALD backgrounds have been included, through their written, visual or audio representations of their voice in stories, comments, and clips in the official digital media created and curated by the NSW Department of Education.

Before presenting the preliminary findings, we provide an overview of the theoretical framework used to consider the role of digital communication in enhancing home–school relationships in public education.

## Organisational communication theory, practices of listening and the public education system

In their account of organisational communication theory, Harris and Nelson (2008) outline three models of communication which can be mobilised to understand how organisations communicate with external stakeholders which may be applied to government–caregiver communication in the public school context. They distinguish between linear, interactional and transactional approaches. Linear communication models represent a one-way communicative approach, where power relations are more traditional, and feedback is not sought as part of the communicative process. Interactional models facilitate a greater incorporation of external input based on a two-way communicative exchange. Transactional communication goes beyond a two-way approach to recognise the ‘complex, dynamic, irreversible, ongoing, contextual, and simultaneous’ nature of communication (Harris and Nelson 2008: 17). The transactional approach to communication provides a greater account of how organisational communication needs to be adaptable to deal with external dynamics that might impact upon the reception of messages and to provide effective opportunities for feedback loops in the diverse array of contexts that impact upon the communication process. The transactional approach to using online and social media can create greater opportunities for members of staff, students and their families to recognise themselves as part of the education system and school community. As they share the experience of engaging with content via online and social media, they may begin to feel as if they share common values and belonging where their voices are valued and included.

As Copeland and de Moor (2018) explain, organisations are able to create a ‘cycle of trust’ in their communications by (1) selecting storytellers who truly represent the stakeholders they tell stories about or perspectives they adopt along with topics that resonate with stakeholders (*legitimacy*), (2) ensuring *authenticity* of voice (vs token participation), (3) enabling the weaving together of stories that represent the diverse community (*synergy*) and (4) providing an open space whereby one social innovation can lead to another (*commons*). If there is a lack of representation or assumptions about cultural norms within the community, particular stakeholders may feel alienated or excluded or feel the content is not relevant to them.

As with all organisations, educational bodies are becoming increasingly active in producing their own media through websites and the use of social media and it is through institutional media that caregivers may gain their greatest connection to the organisation and community that underpins it. It is through organisational media that audiences can develop or maintain a sense of connection and belonging as well as experiment with their identity through relating to others depicted as being part of the community (cf. Anderson, 2006; Hall, 1992: 293–297; Nelson, 1999; Spickard, 2001). For migrants it may be their primary way of fashioning a new identity for themselves in their new place of residence. The question of representation is therefore one of high importance when examining issues of inclusion and cultural belonging when it comes to the mandate of governments and the public education system as the way that official media, including websites, social media and podcasts frames stakeholders from CALD backgrounds will have a flow-on effect as to whether CALD communities will feel included or that their needs are met. As Devine (2009) highlights, it is important to recognise the diversity of migrant student experiences, particularly given that migrant groups are not homogeneous, but represent a multiplicity of values, knowledge and histories. If the official digital spaces do not provide a space to engage in dialogue, stakeholders may feel more comfortable engaging in private or more niche conversations with members of their existing communities, meaning that school organisations may be excluded from the conversations happening among migrant families, with further ramifications in terms of broader social inclusion, cohesion and marginalisation.

When conceptualising school administrators’ mediated representations of identity and considering how they might be able to incorporate a transactional approach to communicative exchange, the concepts of the politics of listening and voice (Bickford, 1996; Couldry, 2006, 2010; Dreher, 2009, 2017; Lacey, 2013) can be used. In media studies, listening has been conceptualised as a fundamental component of the expression of voice within society, and a number of scholars have investigated voice and listening in relation to marginalised groups in traditional and new media contexts, including institutions’ own media platforms (Dreher, 2009; Edwards, 2018; Flew and Panjaitan, 2019; Macnamara, 2013, 2016; Muscat, 2019; Sun, 2012). Scholars have also highlighted the potential for digital and social media to create spaces for engagement and provide a more democratic way for citizens to share experiences (see, e.g. Bruns, 2008; Jenkins et al., 2003). In his work on voice, Couldry (2010) argues the issue is not just one of how technologies enable the expression of voice, but rather how voice is *valued* and listened to by institutions. As Flew and Panjaitan (2019) indicate in their study of voice and Indonesian local government digital communications, the potential to express voice is not always realised and developed into inclusive and impactful participation in the government decision-making processes. It is only when the government actually listens to the voices and attributes value to them that the diverse voices have any meaningful civic impact. While the impact on the decision-making processes cannot be ascertained through an analysis of digital media alone, an organisation’s facilitation of the voices of diverse stakeholders through inclusive representations is important for understanding decisions made around who is chosen to be included within the posts, how they are presented for public viewing, and the value they place on diverse views.

In the educational context, the politics of listening and the transactional model can be mobilised to develop an account of the ways in which marginalised voices are amplified and incorporated into governmental communication. In her account of the politics of listening and media, Lacey (2013) emphasises that there is ‘an ethical obligation to listen out for otherness, for opinions that challenge and clash with one’s own, for voices that take one out of one’s comfort zone’ (p. 195). Here, the practice of ‘listening out’ remains analytically distinct from ‘listening in’, where ‘listening out’ is ‘an attentive and anticipatory communicative disposition’ with political significance, while ‘listening in’ is ‘a receptive and mediatized communicative action’ (Lacey, 2013: 8). Within the context of digital media and schooling, practices of ‘listening out’ can be seen through the markers of opening up organisational dialogic spaces for the expression of diverse values, voices, and experiences. How these diverse opinions and experiences are then recognised and amplified in official communication can demonstrate active efforts to politically engage with diversity and not simply respond to stakeholders in an interactional manner via social media comments. As Schnieder and Arnot (2018) indicate in their work on education, school ‘communication systems need to offer opportunities for dialogue, recognise the value of “Others” and be adaptable to change’ (p. 260), and political practices of ‘listening out” can help to create these opportunities. If the institutions that claim to be inclusive themselves do not ‘listen out’ for diversity, the risk is that *audiences* will choose to ‘listen out’ to alternative sources and focus more on other conversations that give recognition to diverse voices that may be missing in mainstream coverage, as Muscat (2019) found in her study of culturally diverse audiences and Australian mainstream news.

In this article, we analyse the NSW Department of Education’s digital media considering how CALD communities are represented as well as how listening has been facilitated and incorporated as part of the governmental communications during the COVID-19 crisis. Key questions relating to engagement with stakeholders online include (1) What digital media spaces and which languages are being used to share important information with CALD stakeholders, especially parents and caregivers? (2) How were CALD communities represented in the official online and social media of one of the world’s largest government-run educational institution during a critical period impacted by COVID-19? (3) To what degree were external stakeholders (especially caregivers) afforded a voice in government online and social media with regards to their children’s education, particularly those from historically marginalised language backgrounds? (4) What can be learnt from the findings that will help contribute to developing a set of best-practice principles for government–stakeholder communication in CALD contexts? By analysing the online and social media of the NSW Department of Education during COVID-19, this article delves into some of the innovations, challenges and possibilities around government–stakeholder communication in the context of a public education system, with the aim of spearheading further research that will lead to a baseline for best-practice engagement with multicultural and multilingual stakeholders online.

## Methodology

The data collected for this article began with a process of media mapping. The different publicly available official public online and social media platforms used by the NSW Department of Education were first canvassed to identify how they were actively attempting to engage with stakeholders including families, students and teachers/staff, between January 2020 and July 2020, focusing on the period of the start of March to the end of May when COVID-19 had a particularly major impact on NSW schools. We included analysis of the Department’s website, Facebook page, Twitter account and YouTube account as well as the ‘Every Student’ podcast, all of which were active before COVID-19. The Department had since 7 February 2018 also dabbled in WeChat in Chinese language, which is an interesting development for this research but as the content was minimal and has been focussed on overseas engagement with ‘registered agents, Chinese parents and students interested in studying in Sydney or other regions of NSW, Australia’ (NSW Government Schools on WeChat, n.d.) rather than families already living in NSW, we have not included this data in this article.

Media reports mentioning the NSW Department of Education during the set period were also monitored through Factiva. All media releases from this time period were collected manually from the NSW Department of Education’s website. QSR NVivo NCapture was utilised to capture social media posts from the Department’s official Facebook, Twitter and YouTube accounts. This data set was refined through purposive sampling via the use of search terms to focus on posts with any reference to cultural and linguistic diversity in the written or spoken text or images. Only posts/articles/podcasts that related to cultural/linguistic content were included in the final analysis. These posts were analysed through a qualitative thematic content analysis to generate a broad understanding of how the Department might be creating digital spaces for diverse communities to express their voice, and how the representation of these groups online might point to ways in which the Department is listening to and recognising the needs of these communities as well as areas for enhanced engagement.

While audience reactions and comments on selected social media posts were included in the analysis to get a quantitative sense of the level and type of engagement around key posts relating to culture and language, the primary focus of this study was an analysis of the Department’s content, with a focus on how they are representing their diverse stakeholders as well as any efforts to encourage interaction and engagement to elicit the voices of stakeholders. As such, apart from audience reactions on social media, this article does not focus on audience analysis.

## Findings

### Website, media releases and media relations

Being a less interactive platform, the NSW Department of Education website adopts a linear, one-way approach, focused on disseminating important information rather than using this space to engage with stakeholders. Multilingual translations of important documents (e.g. on attendance, on enrolment) are shared in 36 written languages. Interestingly, only traditional Chinese, commonly used in Hong Kong and Taiwan, is used and not simplified Chinese, which is used in Mainland China, which a major place of origin for Chinese speaking migrants at present. There are also audio recordings in 36 languages (including Mandarin and Cantonese) informing parents on the interpreting service available and the number to call (Translated Documents, n.d.). While the government has provided the resources, it may be up to schools themselves to share the relevant materials as needed with their own communities as they links may not be easy to locate by the people who need them from the website alone.

The NSW Department of Education also releases media releases on a daily basis via its website (News, n.d.). While one media release during the COVID-19 period when parents were encouraged to keep their children at home indicated that schools would ‘communicate directly with parents on what learning options are available using their communication methods such as the school website, newsletters, emails and other online tools’ (Latest Schools Info, 2020), it did not share any specific indicators of how parents or students from LBOTE or EAL/D backgrounds would be particularly engaged in this process. However, media monitoring indicated that Department spokespeople were discussing these issues with the media. For instance, in one Australian Broadcasting Corporation (ABC) news report, the Department representative noted that schools worked actively to loan devices and that they acknowledged the additional language problem that some parents faced (Yang et al., 2020). They noted that the Department had developed advice for learning from home for parents and carers in 35 languages, which was available via the Learning from Home Hub and translated documents page. They also noted that the free Translating and Interpreting Service remained available to assist parents and carers with enquiries, though the same report indicated that there was limited access to interpreters due to lockdown restrictions. The Department also explained that it was targeting community language broadcasters to advise parents on how to use the learning from home resources, but there was no mention of the role of social media to engage with multilingual communities. SBS also engaged a Department spokesperson who explained that 4200 devices including computers, modems and 4G Internet dongles were provided to in-need students with more to follow. However, the priority in the first instance was Year 12 students and disadvantaged students in rural and remote schools and no mention was made of the needs of EAL/D families in Sydney (Truu, 2020).

### Podcast

The NSW Department of Education runs ‘The Every Student’ podcast, which is hosted by Mark Scott, who has headed the Department as its Secretary since September 2016. The former managing director for the ABC, Australia’s national broadcaster, interviews innovative educators across the state, and these have been shared twice a month via the Department’s website and other podcasting platforms like iTunes since April 2019. Scott has drawn on his leadership and experience in both the communications and education sectors to take the NSW Department of Education into a new phase of online and digital media engagement as he did with the ABC (The Secretary, 2019).

While focusing more on sharing ideas among educators rather than parents, one interview during the period of analysis, on 6 April 2020, indicated that Scott and the Department were thinking about issues of language and equity. He chose to interview Brad Lanham, the acting principal of Canley Vale Public School, a school in Sydney’s South-West with 97% EAL/D students. During the interview, Lanham explained the strategies his school was using to engage with families, particularly with parents who may not only speak limited English but also be illiterate in their home language. Strategies included using community liaison officers and language teachers to audio-record translations of letters in home languages which parents could listen to by scanning the QR code on their mobile phones. Following this they made phone calls to parents in their home languages, which Lanham noted ‘did have an impact’ and was ‘most vital’. He noted that it was ‘sending out bulk SMSs that ensured greater engagement throughout the website’. Lanham also spoke about ensuring that students have the direct email address of their teacher and assistant principal attached to their reports to encourage open communication. Overall, he highlighted the importance of the community liaison officers and community language teachers who translate between the classroom teachers and home and that it was important to not just send translated letters home but to ‘keep that personal aspect’ (Every Student Podcast: Brad Lanham, 2020).

### Social media – Facebook

The NSW Department of Education has overall taken most initiative towards engagement with community stakeholders via social media, particularly on Facebook with numerous posts each day. It is clear that the Department is aiming to be conversational, engaging and celebratory, with a mix of informational and uplifting stories shared via video, image and text, with the same videos also posted to its YouTube channel. It has also been prepared to deal with critical feedback from parents. For example, one post on 19 May, in which the plan to return to school 5 days a week immediately rather than gradually increasing the days from 1 day a week resulted in over 1600 reactions, 1700 comments and 1700 shares. The Department responded actively to the concerns to manage the criticism. A day later it generated over 900 positive reactions, over 350 comments and over 70 shares for uplifting posts with images of students excitedly returning to school.

Although we did not see any specific evidence of engagement with the EAL/D community via Facebook during the period of analysis, just after the return to regular face-to-face teaching on 29 May, acting principal of Canley Vale Public School, Brad Lanham (noted above for his podcast interview with Mark Scott), was featured in the Department’s Facebook feed through a re-posting of a video sharing his first ‘culinary adventure’ for the ‘Canley Vale Cooking Show’. The post introduced the series in which he would be ‘cooking some cultural dishes with members of the school community, while ALSO trying out his language skills – starting with Cambodian BBQ Beef and Khmer!’. In the clip, Mrs Ly became the ‘teacher’ of the school leader, with the entire exchange in Khmer, including Mr Lanham’s awkward but admirable attempts to speak in Khmer too. With subtitles in English, such a video worked to reverse the ordinary power dynamics between the school leader and the members of the school community, creating a democratic and inclusive feel. The post generated positive feedback on the Department’s Facebook page with over 100 likes and 16 positive comments (as of 14 June) encouraging this genuine attempt at cross-linguistic exchange. One respondent posted ‘Sir you speak Khmer better than my Australian/Khmer son’, while another stated that ‘This should have happened during the first Vietnamese boat arrivals and the arrival of Khmer and Lao refugees but better late than never. A great initiative! – promotes cultural values in all ways A great example to other schools’.

After this post was shared by the NSW Department of Education, which validated the approach of the school towards being culturally and linguistically inclusive, Canley Vale Public School re-posted the same video to their own school Facebook site for a second time. This re-posted content was accompanied with the message ‘Wow! Mrs Ly’s cooking class has been shared on the NSW Department of Education Facebook page. Great work, Mrs Ly’. It received 58 positive reactions as well as four comments which appear to have been from EAL/D parents, including one with a picture and a comment ‘Congrats’ in Khmer and English. These comments and reactions provide a glimpse into understanding the value of incorporating diversity into school communication, and it is through such representations that the voices of multicultural groups are able to be acknowledged. Indeed, the school demonstrated efforts to engage with a range of different CALD groups within the community beyond this particular post with the Khmer community. Mr Lanham was featured on the NSW Department of Education’s Facebook account again on 12 June cooking Vietnamese rice paper rolls with Mrs Mai, with both Mr Lanham and Mrs Mai speaking Vietnamese throughout the entire clip. Significantly, the original ‘Canley Vale Cooking Show’ videos which included Cantonese, Arabic and Mandarin episodes, posted to the Canley Vale Public School Facebook page have attracted far greater engagement within the school community page than on the NSW Department of Education Facebook site, with the Vietnamese video attracting 2400 views and the Khmer video attracting 2300 views, no doubt enhanced by the extra celebrity status afforded through validation by the NSW Department of Education. The Arabic and Mandarin editions also had over 1000 views at the time of writing. This one school’s multilingual culinary show was the most innovative example of cross-linguistic engagement we saw during the COVID-19 period that was endorsed by the Department on its own social media.

## Discussion

As the above examples show, the NSW Department of Education has paid some attention to its role in engaging with parents from LBOTE and EAL/D backgrounds and there are positive instances of effective engagement across languages, or rather validation of these cross-cultural and cross-linguistic experiences as a normal part of the educational community life. This could be seen through the podcast episode with the acting principal of Canley Vale Public School as well as via the Department’s Facebook page, which was the most active example of an attempt to engage in a two-way interactional manner with stakeholders in a public forum. By re-sharing posts from Canley Vale Public School, the Department demonstrated the potential for LBOTE and EAL/D engagement in an inclusive manner in the context of their ‘owned media’. The selected posts provide crucial insight into instances where a local school has opened up their social media spaces to feature diverse migrant voices and experiences, while the Department’s re-sharing accords further recognition and validation of such diverse voices, experiences and languages as part of the broader public school community.

However, these examples of cross-cultural and cross-linguistic engagement were not largely representative of the types of posts featured on the Department’s Facebook feed during this period and in this context and only relate to one school. It is the case, however, as outlined above, that Sydney’s schools are incredibly culturally diverse and while Canley Vale Public School may have a particularly high percentage of CALD and EAL/D students, it is not unusual to have over 50% of students from a CALD background. Thus, there is room for further normalising these kinds of interactions through more frequent inclusion particularly through social media which lends itself well to ‘social’ interactions, but can also be further considered in the context of the institutional website to allow for easy access to materials through multilingual webpages (not just documents) and multicultural and multilingual storytelling beyond the more ‘linear’ provision of information and announcements which remains important.

The Department’s stance of actively demonstrating a desire to learn from the educators ‘on the ground’ as the podcasts and shares of Mr Lanham’s culinary adventures indicate, demonstrates an interest in practices of ‘listening out’ to the values and experiences of diverse ‘Others’ and to opening oneself up to cultural and linguistic difference. Through these re-posts and interviews, the NSW Department of Education not only has the ability to acknowledge the good work particular school communities are doing, but to start to accord recognition to the variety of migrant communities’ values, knowledges and histories. Although these posts do not explain the inclusion of cultural and linguistic diversity around more consequential decisions regarding school operations, it nonetheless provides a foundation for understanding how the Department might further develop practices of ‘listening out’ to diverse stories and experiences to enhance social cohesion. As one stakeholder’s response to the post highlights, these organisational efforts can be contextualised by the school community within the broader history of migrant experiences in Australia and elucidate the significance of being accorded value through the amplification of traditionally ‘Othered’ voices and identities. The value is not simply on being receptive to migrant communities and ‘listening in’ and responding to their comments, but also for the Department to develop ongoing practices of ‘listening out’ for those values, opinions and knowledge that might clash with the established ways of doing things within the school system.

## Conclusion

This article has raised questions about the representation and voice of parents and caregivers particularly from LBOTE and EAL/D backgrounds in the public education system, and has provided some positive examples of how this can be done via online and social media, via the example of the NSW Department of Education at a time of increased need for online home–school communication as a result of COVID-19. The study has also indicated that there is room for more proactive engagement with CALD stakeholders by such government bodies. If government agencies wish to enhance a sense of belonging to meet their inclusion mandate and actively embrace the challenge it is important that their online and social media communications positively include, represent and engage with stakeholders across cultural and linguistic divides. To develop a more democratic use of digital and social media, practices of ‘listening out’ need to be incorporated as part of a more sustained community effort to engage with diverse values, experiences, and knowledge. The examples discussed here highlight solid foundations, yet for these exchanges to not only be symbolic, the voices and experiences of parents and caregivers from these culturally diverse backgrounds need to be amplified into organisational decision-making.

This article paves the way for further research on the topic of government communication with CALD stakeholders, particularly in the education sector. To complement this study, audience research would be of great interest to better understand the attitudes, behaviour and needs of stakeholders of diverse cultural and linguistic backgrounds in the public education system, and a more extensive audience study including surveys and interviews with caregivers is the focus of the next phase of our research project. This research will also consider the unofficial use of social media by parents and caregivers to discuss issues around their children’s schooling, including the use of predominantly non-English language platforms like WeChat, which has ‘almost ubiquitous use’ among Chinese migrants from the People’s Republic of China (Sun, 2016, 2019). Such research would likely provide illuminating insights into the discussions around the NSW public schooling system from a migrant perspective. The broader project will also canvass the views of stakeholders in government agencies like the NSW Department of Education who are responsible for providing communications with diverse publics, including social media teams and website developers, as well as staff within individual schools who are responsible for communication with parents and caregivers to learn more about the opportunities and challenges. Applied research in this area, in partnership with government education departments and individual schools, could be utilised in a way that may help to establish a set of principles and guidelines to be used to better engage with stakeholders of a diverse and changing demographic background.
